# Supplementary material for: Scaling up drug combination surface prediction
Source: Brief Bioinform. 2025 Mar 13;26(2):bbaf099. doi: 10.1093/bib/bbaf099 (PMC11904408; doi:10.1093/bib/bbaf099)
Supplement: supplementary_bbaf099 [file supplementary_bbaf099.pdf]

Scaling up drug combination surface prediction
Supplementary results

Riikka Huusari, Tianduanyi Wang, Sandor Szedmak, Diogo Dias,
Tero Aittokallio, Juho Rousu

Main results tables

We give the results displayed in Figures 3 and 4 in the main paper in tabular format in Tables 1 (Figure 3) and 2.

Table 1: Results of main paper's Figure 3. Each cell is formatted as: "mean ± standard deviation (p-value)". The statistical testing has been performed as a comparison to comboKR 2.0; thus there are no p-values for those results.

	Method	New combo	New drug	New cell line
Jaaks	comboKR 2.0	0.894 ± 0.001 (-)	0.894 ± 0.004 (-)	0.887 ± 0.010 (-)
	comboKR	0.890 ± 0.001 (1.0×10^{0})	0.841 ± 0.007 ($< 10^{-20}$)	-
	C.LTR mv	0.831 ± 0.004 ($< 10^{-20}$)	0.828 ± 0.003 ($< 10^{-20}$)	0.812 ± 0.006 ($< 10^{-20}$)
	Baseline	0.884 ± 0.001 (1.0×10^{0})	0.885 ± 0.004 (1.0×10^{0})	0.881 ± 0.011 (1.0×10^{0})
NCI-ALMANAC	comboKR 2.0	0.902 ± 0.000 (-)	0.904 ± 0.003 (-)	0.897 ± 0.003 (-)
	comboKR	0.900 ± 0.000 (4.1×10^{-9})	0.889 ± 0.003 ($< 10^{-20}$)	-
	C.LTR mv	0.881 ± 0.002 ($< 10^{-20}$)	0.880 ± 0.005 ($< 10^{-20}$)	0.859 ± 0.007 ($< 10^{-20}$)
	Baseline	0.902 ± 0.000 (1.2×10^{-3})	0.904 ± 0.003 (3.3×10^{-4})	0.897 ± 0.003 (1.3×10^{-3})
O'Neil	comboKR 2.0	0.939 ± 0.001 (-)	0.937 ± 0.002 (-)	0.937 ± 0.007 (-)
	comboKR	0.938 ± 0.001 (1.0×10^{0})	0.907 ± 0.002 ($< 10^{-20}$)	-
	C.LTR 1v	0.902 ± 0.004 ($< 10^{-20}$)	0.866 ± 0.002 ($< 10^{-20}$)	0.826 ± 0.013 ($< 10^{-20}$)
	C.LTR mv	0.909 ± 0.002 ($< 10^{-20}$)	0.869 ± 0.008 ($< 10^{-20}$)	0.838 ± 0.007 ($< 10^{-20}$)
	Baseline	0.930 ± 0.001 (2.8×10^{-7})	0.929 ± 0.002 (4.2×10^{-5})	0.931 ± 0.007 (3.8×10^{-3})

Table 2: Results of main paper's Figure 4. Each cell is formatted as: "mean ± standard deviation (p-value)".

	Method	New combo	New drug	New cell line
Bliss	comboKR 2.0	0.929 ± 0.004 (1.1×10^{-2})	0.928 ± 0.004 (3.7×10^{-3})	0.926 ± 0.011 (2.2×10^{-3})
	comboKR	0.922 ± 0.003 (1.6×10^{-1})	0.857 ± 0.010 (8.2×10^{-5})	-
	DS	0.567 ± 0.010 ($< 10^{-20}$)	0.560 ± 0.017 ($< 10^{-20}$)	0.474 ± 0.022 ($< 10^{-20}$)
	MM	0.783 ± 0.010 (2.3×10^{-3})	0.690 ± 0.016 (6.0×10^{-13})	0.530 ± 0.022 ($< 10^{-20}$)
	C.LTR 1v	0.877 ± 0.007 (1.0×10^{0})	0.806 ± 0.007 (5.4×10^{-1})	0.700 ± 0.020 (9.4×10^{-1})
	C.LTR mv	0.895 ± 0.004 (1.0×10^{0})	0.815 ± 0.020 (2.3×10^{-1})	0.742 ± 0.010 (2.8×10^{-2})
	Baseline	0.912 ± 0.004 (1.0×10^{0})	0.913 ± 0.004 (1.0×10^{0})	0.913 ± 0.011 (1.0×10^{0})
Loewe	comboKR 2.0	0.935 ± 0.003 (1.2×10^{-2})	0.932 ± 0.003 (1.0×10^{-2})	0.932 ± 0.007 (4.1×10^{-3})
	comboKR	0.930 ± 0.003 (1.8×10^{-2})	0.875 ± 0.004 (2.8×10^{-6})	-
	DS	0.286 ± 0.008 (9.5×10^{-3})	0.246 ± 0.017 (5.6×10^{-4})	0.111 ± 0.032 (6.2×10^{-17})
	MM	0.628 ± 0.010 (3.9×10^{-7})	0.455 ± 0.024 (1.2×10^{-3})	0.202 ± 0.033 (2.6×10^{-12})
	C.LTR 1v	0.891 ± 0.007 (1.0×10^{0})	0.831 ± 0.011 (1.0×10^{0})	0.763 ± 0.019 (2.3×10^{-1})
	C.LTR mv	0.906 ± 0.002 (1.0×10^{0})	0.836 ± 0.018 (7.9×10^{-2})	0.789 ± 0.013 (3.0×10^{-1})
	Baseline	0.919 ± 0.003 (1.0×10^{0})	0.918 ± 0.003 (1.0×10^{0})	0.920 ± 0.007 (1.0×10^{0})

Overall results

We present here overall results w.r.t Spearman correlation (Figure 1), as well as with Pearson correlation over the calculated Bliss and Loewe synergy scores (Figures 2 and 3).

Density plots

Figure 4 shows example density plots of the results with one training-test split on O'Neil dataset.

Pairwise comparison of predictions' RMSE to groundtruth

We show pairwise comparison of the methods in Tables 3, 4 and 5. In these tables, for each method, for each predicted surface, the root mean squared error (RMSE) has been computed with respect to the groundtruth measurements. For each pair of methods, the tables report how often (i.e. on how many surface in the test set) did one method outperform the other.

Ablation results

In addition to the Pearson correlation results on the comboKR 2.0 variants presented in the main paper, we here display the Spearman correlation results in Figure 5. Similarly, results from Bliss and Loewe synergy scores are shown in Figures 6 and 7.

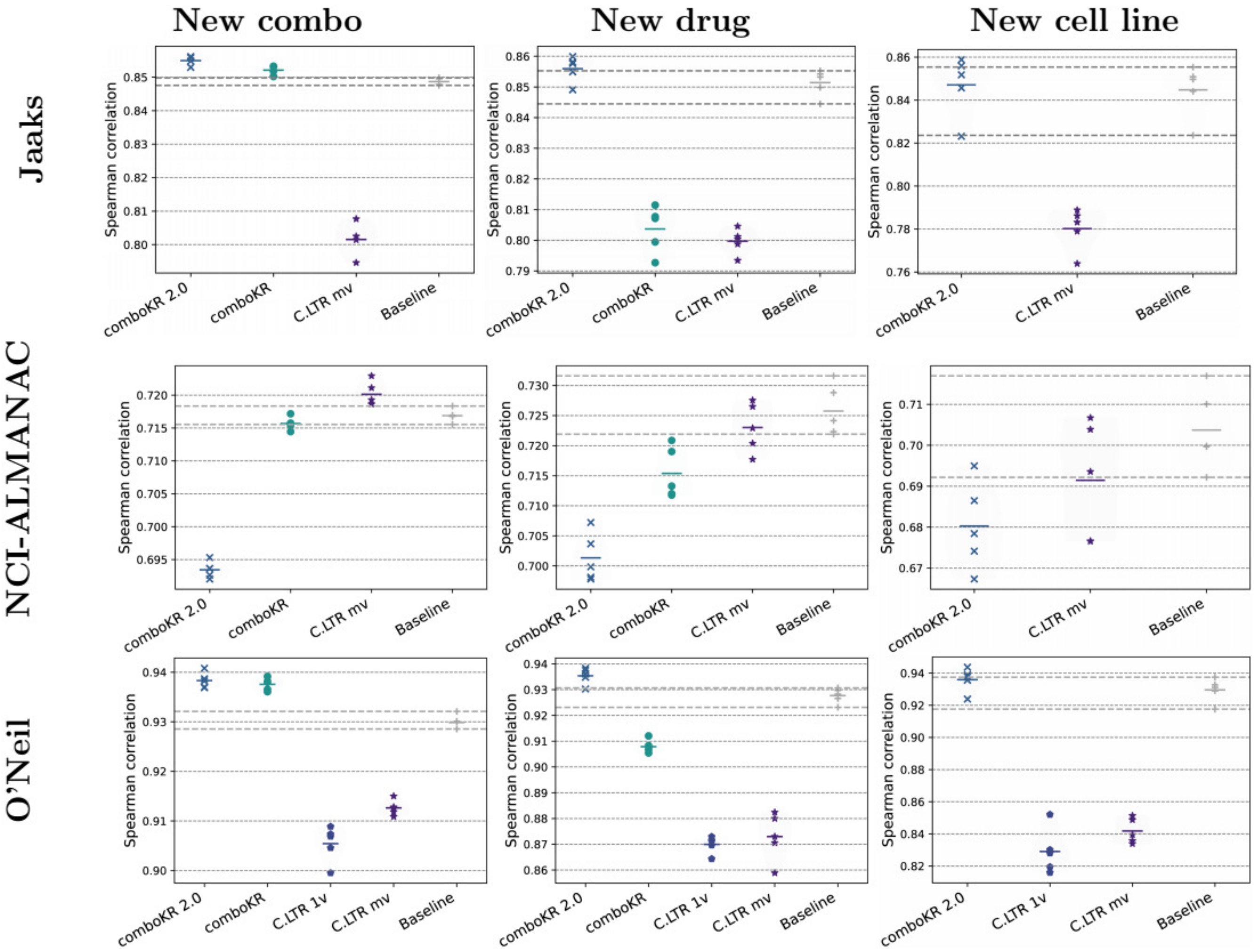

Figure 1: Spearman correlation results of the various methods on the three datasets, on the predicted response values. The original ComboKR model is trained cell-by-cell manner, while the other methods are over multiple cell lines: thus, the original formulation is not applicable in the new cell line scenario.

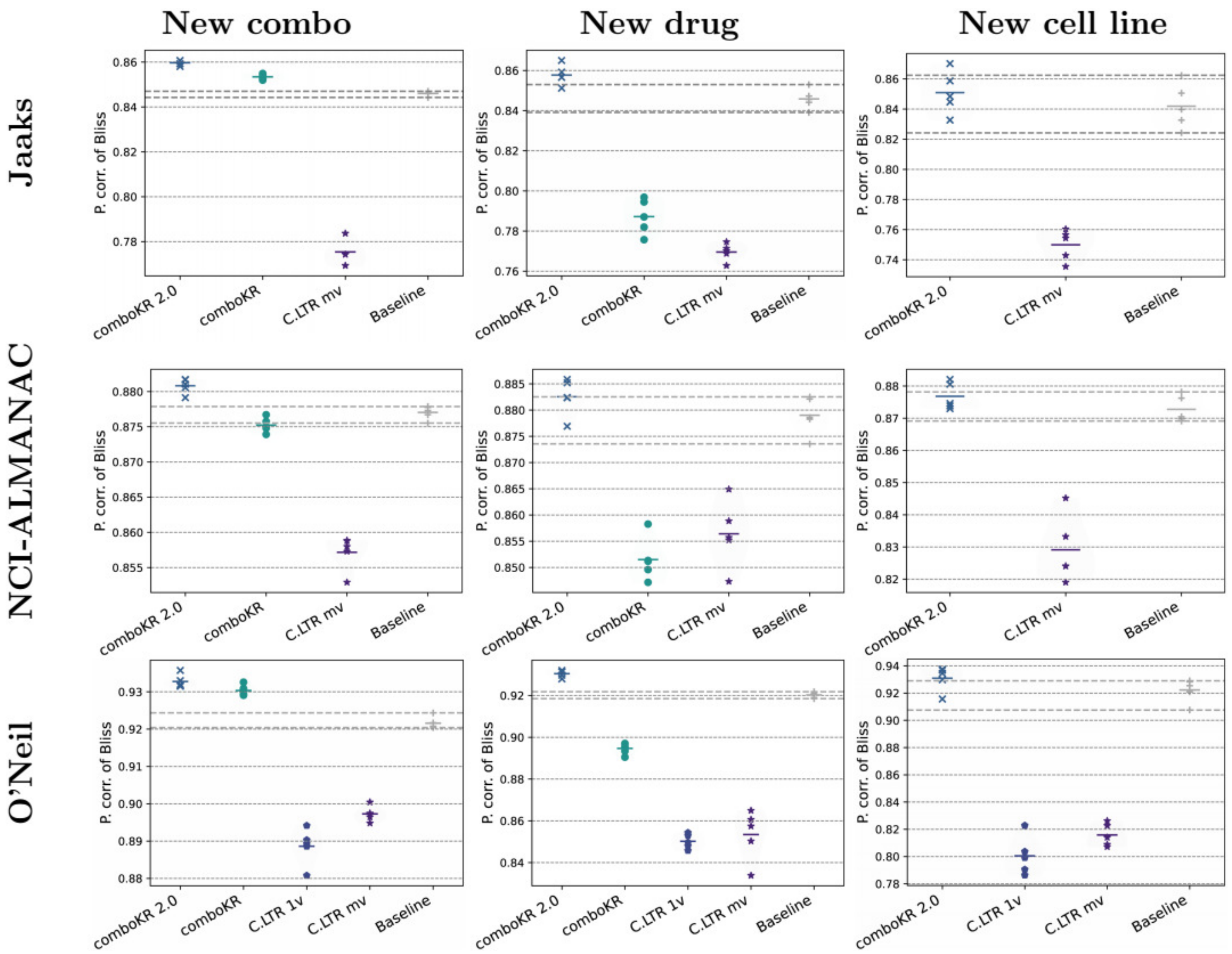

Figure 2: Pearson correlation results of the Bliss synergy scores calculated from the predictions of the various methods on the three datasets, compared to the synergy scores computed from measured ground truth response values. The original ComboKR model is trained cell-by-cell manner, while the other methods are over multiple cell lines: thus, the original formulation is not applicable in the new cell line scenario.

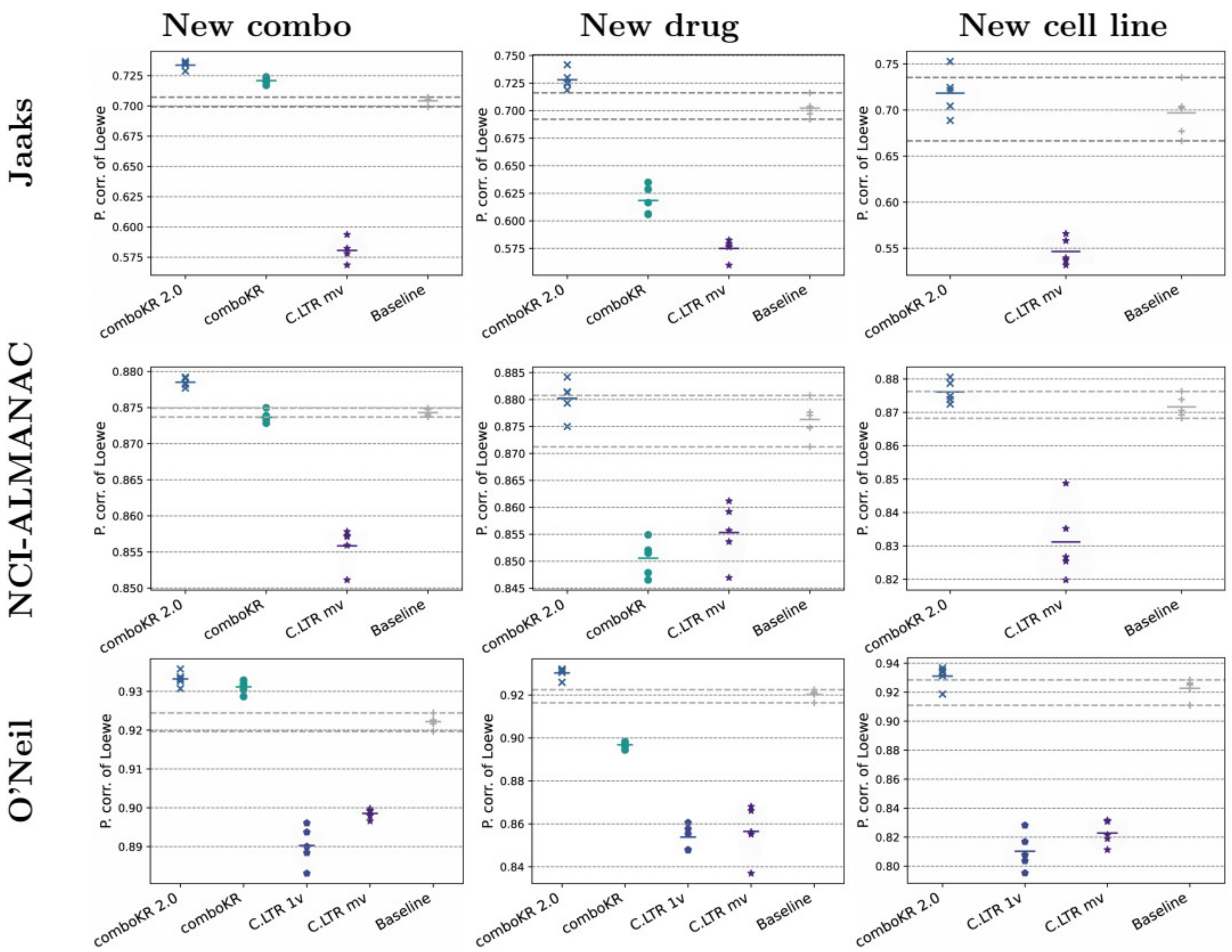

Figure 3: Pearson correlation results of the Loewe synergy scores calculated from the predictions of the various methods on the three datasets, compared to the synergy scores computed from measured ground truth response values. The original ComboKR model is trained cell-by-cell manner, while the other methods are over multiple cell lines: thus, the original formulation is not applicable in the new cell line scenario.

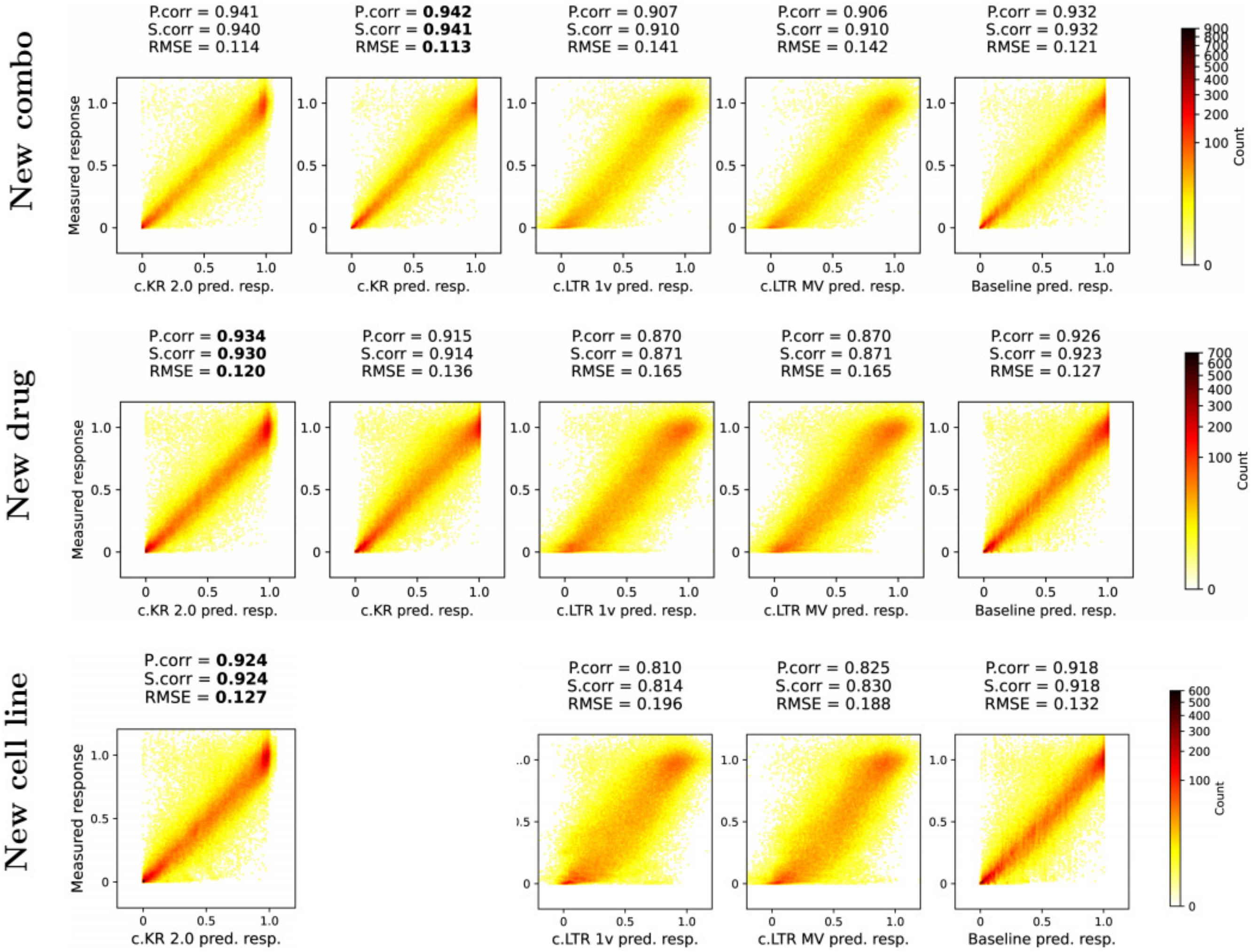

Figure 4: Density plots of the results in the different predictive scenarios with the O'Neil dataset. The bold font in titles highlights best performance.

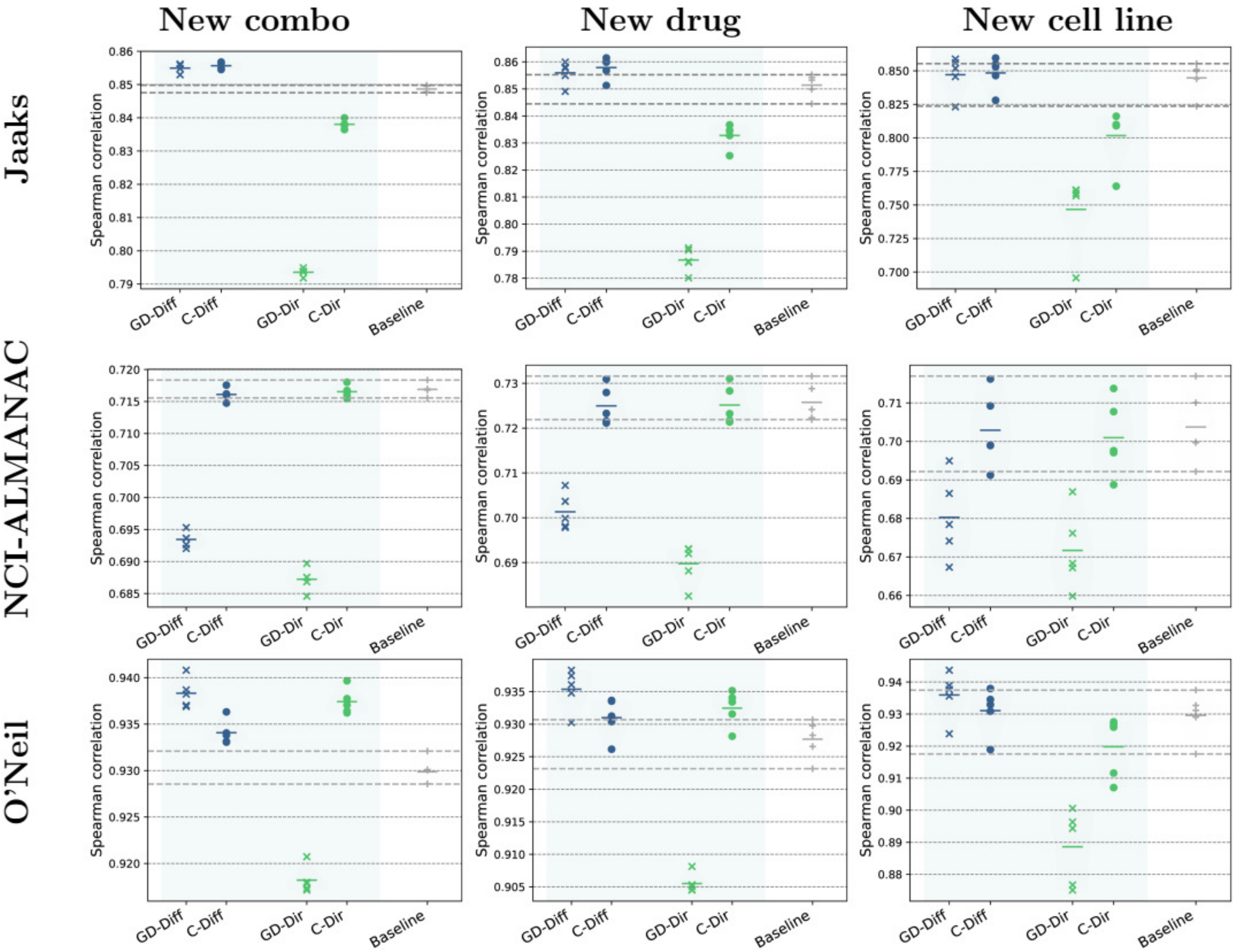

Figure 5: Spearman correlation results of the comboKR 2.0 variants on the three datasets, on the predicted response values.

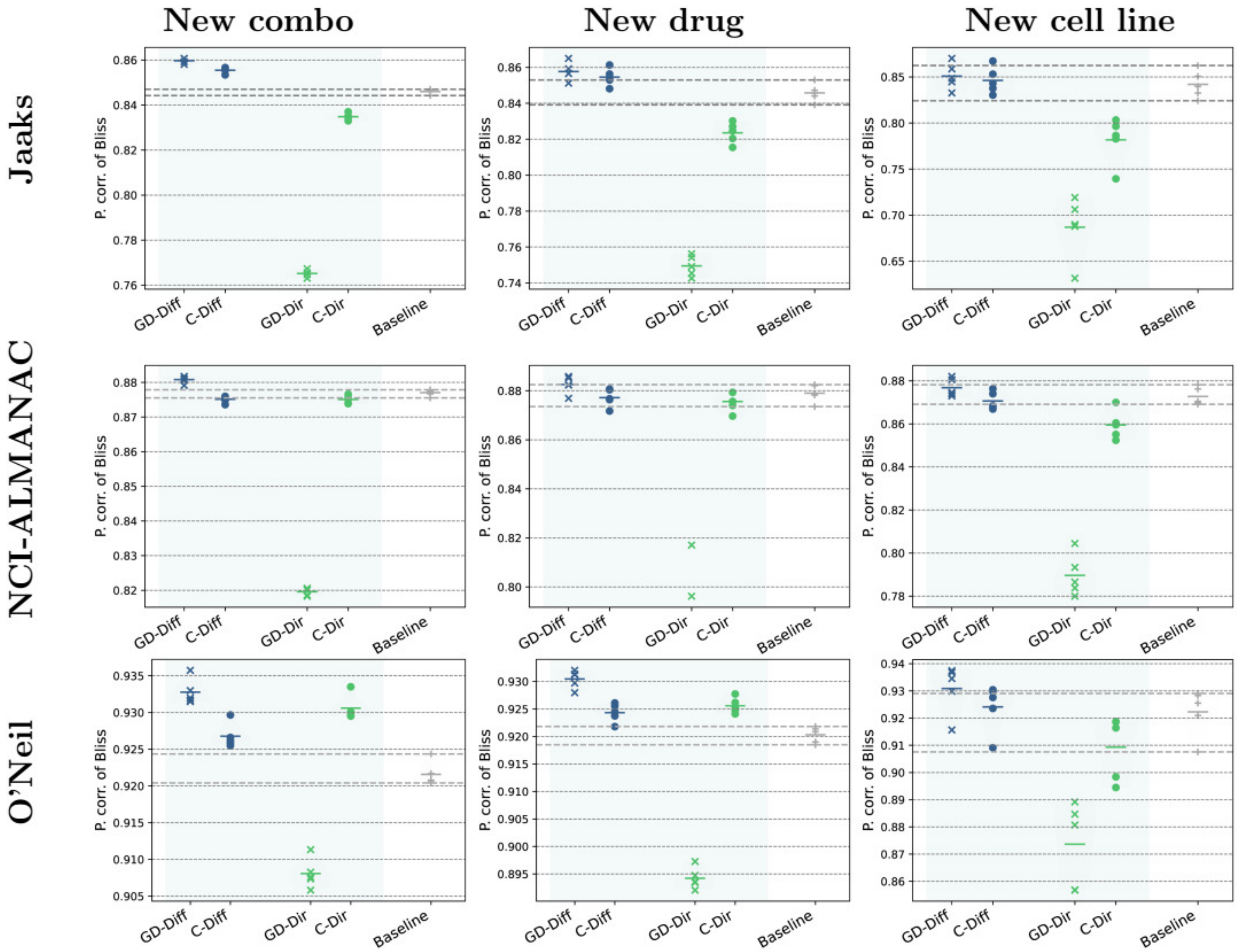

Figure 6: Pearson correlation results of the Bliss synergy scores calculated from comboKR 2.0 variants' results and groundtruth data, on the three datasets.

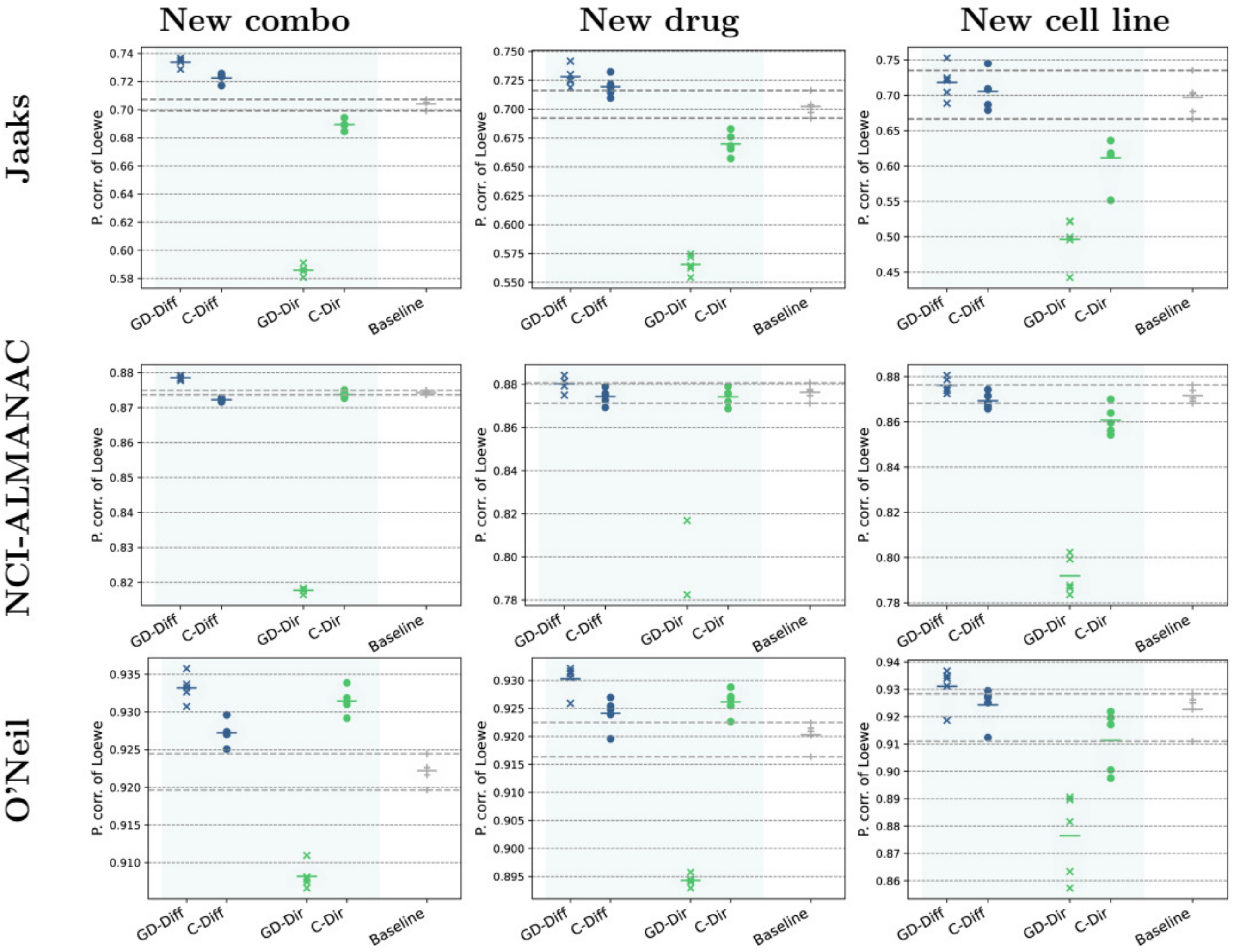

Figure 7: Pearson correlation results of the Loewe synergy scores calculated from comboKR 2.0 variants' results and groundtruth data, on the three datasets.

Table 3: Jaaks data. A cell in the table tells how often method on the row had smaller RMSE to groundtruth than the method on the column; darker rows and lighter columns indicate method performed better than others.

New combo

	cKR 2.0	cKR	cLTR MV	Baseline
cKR 2.0		49.8%	68.5%	53.4%
cKR	50.2%		67.9%	51.8%
cLTR MV	31.5%	32.1%		33.0%
Baseline	46.6%	48.2%	67.0%	

New drug

	cKR 2.0	cKR	cLTR MV	Baseline
cKR 2.0		62.8%	68.4%	51.4%
cKR	37.2%		55.8%	38.0%
cLTR MV	31.6%	44.2%		32.0%
Baseline	48.6%	61.9%	68.0%	

New cell line

	cKR 2.0	cLTR MV	Baseline
cKR 2.0		69.1%	50.2%
cLTR MV	30.9%		30.8%
Baseline	49.8%	69.2%	

Table 4: NCI-ALMANAC data. A cell in the table tells how often method on the row had smaller RMSE to groundtruth than the method on the column; darker rows and lighter columns indicate method performed better than others.

New combo

	cKR 2.0	cKR	cLTR MV	Baseline
cKR 2.0		43.8%	61.7%	50.1%
cKR	56.2%		63.0%	53.7%
cLTR MV	38.3%	37.0%		37.3%
Baseline	49.9%	46.3%	62.7%	

New drug

	cKR 2.0	cKR	cLTR MV	Baseline
cKR 2.0		51.7%	62.1%	49.2%
cKR	48.3%		58.0%	45.8%
cLTR MV	37.9%	42.0%		36.7%
Baseline	50.8%	54.2%	63.3%	

New cell line

	cKR 2.0	cLTR MV	Baseline
cKR 2.0		68.2%	50.2%
cLTR MV	31.8%		31.1%
Baseline	49.8%	68.9%	

Table 5: O'Neil data. A cell in the table tells how often method on the row had smaller RMSE to groundtruth than the method on the column; darker rows and lighter columns indicate method performed better than others.

New combo

	cKR 2.0	cKR	cLTR 1V	cLTR MV	Baseline
cKR 2.0		46.9%	74.1%	72.4%	61.1%
cKR	53.1%		73.5%	71.9%	57.4%
cLTR 1V	25.9%	26.5%		45.2%	30.0%
cLTR MV	27.6%	28.1%	54.8%		31.5%
Baseline	38.9%	42.6%	70.0%	68.5%	

New drug

	cKR 2.0	cKR	cLTR 1V	cLTR MV	Baseline
cKR 2.0		61.6%	78.5%	77.7%	59.5%
cKR	38.4%		66.5%	65.1%	42.0%
cLTR 1V	21.5%	33.5%		47.9%	24.4%
cLTR MV	22.3%	34.9%	52.1%		25.2%
Baseline	40.5%	58.0%	75.6%	74.8%	

New cell line

	cKR 2.0	cLTR 1V	cLTR MV	Baseline
cKR 2.0		83.7%	83.2%	58.3%
cLTR 1V	16.3%		46.0%	18.3%
cLTR MV	16.8%	54.0%		18.8%
Baseline	41.7%	81.7%	81.2%	
